# A meta-analysis to estimate the “real” placebo effect in juvenile idiopathic arthritis (JIA) trials

**DOI:** 10.1186/1546-0096-9-S1-P192

**Published:** 2011-09-14

**Authors:** E Demirkaya, N Ruperto, R Galasso, A Ravelli, E Palmisani, A Martini, A Pistorio

**Affiliations:** 1 IRCCS G Gaslini, Pediatria II, Reumatologia, PRINTO, Genova, Italy; 2 IRCCS G. Gaslini, Pediatria II, Reumatologia and Dipartimento di Pediatria, Università degli Studi, Genova, Italy; 3 IRCCS G Gaslini, Servizio di Epidemiologia e Biostatistica, Genova, Italy

## Objectives

To quantify placebo effect through a systemic review of juvenile idiopathic arthritis (JIA) trials using placebo as comparator.

## Methods

This study was developed according to the PRISMA statement and pre-specified study selection, eligibility criteria, data extraction, quality assessment, and statistical analysis. Studies with parallel, cross-over or withdrawal design (open label followed by a double-blind placebo withdrawal period) were included in the analysis. Publications were retrieved from MEDLINE, Web of Science, EMBASE, and the Cochrane Controlled Trials Register from 1960 to May 2010, with literature search carried out from February to December 2010. Estimates of the placebo response rate and estimates of the placebo flare rate for the withdrawal trials with 95% Confidence Intervals (CI) were calculated.

## Results

A total of 10 out of 18 trials were included in the final meta-analysis synthesis. In the 5 trials with parallel design the number of responders among the patients randomized to the placebo arms were 76/246 (30.7%) for recent studies with composite scores such as the ACR pediatric 30 criteria with a pooled placebo effect estimate (fixed effect method) equal to 30% (95% CI 24-36%).

In the5 withdrawal design trials the number of flared patients randomized to the placebo arms was 89/123 (72.4%) with a pooled effect estimates (percentages of placebo patients who have a flare during the double blind phase in the placebo group) equal to 74% (95% CI 67-82%).

## Conclusions

This systematic review provides reference data for the mean placebo effect for parallel trials and mean placebo flare rate for trials with withdrawal design.

